# Leisure-Time Physical Activity Among Women of Reproductive Age — United States, 2022 and 2024

**DOI:** 10.15585/mmwr.mm7441a2

**Published:** 2025-12-18

**Authors:** K.A. Kamanani Conklin, Jasmine Y. Nakayama, Miriam E. Van Dyke, Kim G. Harley, Hannah R. Thompson

**Affiliations:** ^1^School of Public Health, University of California at Berkeley, Berkeley, California; ^2^Division of Nutrition, Physical Activity, and Obesity, National Center for Chronic Disease Prevention and Health Promotion, CDC.

**Summary**

**What is already known about this topic?**

Physical activity has numerous health benefits for women of reproductive age. National guidelines include recommendations for both aerobic and muscle-strengthening physical activity.

**What is added by this report?**

In this 2022 and 2024 national survey, an estimated 25.1%, 21.7%, and 6.1% of U.S. women aged 18–44 years reported leisure time physical activity meeting recommendations for both aerobic and muscle-strengthening activity, aerobic activity only, and muscle-strengthening activity only, respectively; an estimated 47.2% reported leisure-time physical activity insufficient to meet either recommendation. Prevalences of aerobic and muscle-strengthening activity varied by race and ethnicity, age, and educational attainment.

**What are the implications for public health practice?**

Addressing possible population-specific barriers to physical activity among women could be an important strategy for improving health outcomes.

## Abstract

Physical activity has numerous health benefits, including for women of reproductive age (18–44 years), among whom it can prevent chronic disease, including osteoporosis, and improve maternal health. Understanding the prevalence of leisure-time physical activity among different sociodemographic groups of women of reproductive age can help guide public health interventions and messaging. Data from the 2022 and 2024 National Health Interview Survey were used to examine the prevalences of self-reported leisure-time physical activity and meeting recommendations in the *Physical Activity Guidelines for Americans, 2nd edition*, among 10,981 women aged 18–44 years by race and ethnicity, age, and educational attainment. Overall, an estimated 25.1% of women aged 18–44 years reported leisure time activity meeting recommendations for both aerobic and muscle-strengthening physical activity, 27.1% reported leisure time activity meeting only the aerobic activity recommendation, and 6.1% reported leisure time activity meeting only the muscle-strengthening activity recommendation. An estimated 47.2% of women reported leisure-time physical activity insufficient to meet either recommendation (including those reporting no leisure time physical activity). Prevalences of reported aerobic and muscle-strengthening physical activity varied by race and ethnicity, age, and educational attainment: higher percentages of younger women (women aged 18–24 years), non-Hispanic White (White) women, and women with higher levels of educational attainment met both recommendations than did older women (women aged 30–34 or 40–44 years), women who are not White, and those with less educational attainment. Given the benefits of physical activity for maternal, physical, and mental health, addressing possible population-specific barriers to physical activity among women of reproductive age could be an important strategy for improving health outcomes.

## Introduction

Physical activity improves health across the lifespan (*1*). Among women of reproductive age,* regular physical activity provides immediate benefits, including improved sleep and mental health (*1*). Aerobic physical activity and muscle-strengthening activity can both improve critical indicators of cardiometabolic health (e.g., blood pressure, body composition,^†^ and glucose metabolism) and lower the risk for debilitating and costly chronic diseases later in life (*1*). Muscle-strengthening activity provides unique benefits, including increased bone strength and density and muscle mass retention during weight loss (*1*). During pregnancy, regular aerobic physical activity can reduce the risk for delivering a low-birthweight infant, gestational diabetes, hypertension, and postpartum depression (*1*,*2*), and muscle-strengthening activity can reduce the risk for low back pain and Cesarean delivery (*2*). However, most U.S. women do not meet the recommendations included in the *Physical Activity Guidelines for Americans, 2nd edition* (*3*), which are for moderate-to-vigorous aerobic physical activity (≥150 minutes per week at moderate intensity, ≥75 minutes per week at vigorous intensity, or an equivalent combination) and muscle-strengthening activities (two or more sessions per week) (*1*). National estimates of physical activity among women of reproductive age stratified by sociodemographic characteristics are unavailable, although studies find that adult leisure-time physical activity generally varies by race and ethnicity, age, and educational attainment (*4*). Ascertaining the prevalences of meeting physical activity guidelines stratified by these factors can identify groups that might most benefit from physical activity–promoting initiatives. Data from the 2022 and 2024 National Health Interview Survey (NHIS) were analyzed to describe the prevalence of meeting physical activity guidelines and participation in leisure-time moderate-to-vigorous aerobic physical activity and muscle-strengthening activity among U.S. women aged 18–44 years by race and ethnicity, age, and educational attainment.

## Methods

### Data Source

NHIS is a continuous cross-sectional household interview survey of the noninstitutionalized, U.S. civilian population across 50 states and the District of Columbia (2022 and 2024 response rates: 48%).^§^ The 2 most recent years with physical activity data (2022 and 2024) were combined to increase precision of estimates, yielding 11,412 female respondents aged 18–44 years. Sensitivity analyses verified that 2022 and 2024 data were comparable, with no substantial between-year differences among demographic groups in prevalences of physical activity sufficiency or of meeting the physical activity guidelines. The 521 (4.6%) respondents for whom data on moderate-to-vigorous aerobic physical activity, muscle-strengthening activity, or educational attainment were missing were excluded, resulting in an analytic sample of 10,891 respondents (weighted to nationally represent 56,087,223 women).^¶^

### Characterization of Physical Activity

Respondents reported participation in leisure-time moderate-to-vigorous aerobic physical activity** and muscle-strengthening activity^††^ and were classified into one of four categories of meeting physical activity guidelines: 1) meeting neither guideline (neither aerobic nor muscle-strengthening activities); 2) meeting both physical activity guidelines; 3) meeting aerobic physical activity guidelines only; or 4) meeting muscle-strengthening physical activity guidelines only.^§§^ Separately, four levels of moderate-to-vigorous aerobic physical activity participation (none, insufficient, sufficient, or high)^¶¶^ and three levels of muscle-strengthening activity participation (none, insufficient, or sufficient)*** were assessed.

### Data Analysis

Prevalence estimates (and 95% CIs) were calculated for physical activity measures. Wald chi-square tests were used to evaluate differences in prevalence estimates of physical activity measures across demographic groups; where appropriate, a Bonferroni correction accounted for multiple tests. Statistical significance was defined as a p-value <0.05; all reported differences are statistically significant. To generate national estimates, analyses in Stata (version 17; StataCorp) used survey weights to account for the complex survey design and nonresponse. This activity was reviewed by CDC, deemed not research, and was conducted consistent with applicable federal law and CDC policy.^††† ^

## Results

### Meeting Physical Activity Guidelines

Overall, an estimated 47.2% of women aged 18–44 years reported leisure-time physical activity that met neither aerobic nor muscle-strengthening physical activity guidelines, 25.1% reported activity that met both physical activity guidelines, 21.7% reported activity that met aerobic physical activity guidelines only, and 6.1% reported activity that met muscle-strengthening guidelines only (Table 1). Non-Hispanic Asian (Asian) women, non-Hispanic Black or African American (Black) women, and Hispanic or Latino (Hispanic) women were less likely to report leisure-time activity meeting both physical activity guidelines (20.4%, 21.4%, and 23.4%, respectively) compared with non-Hispanic White (White) women (27.6%). The prevalence of reporting activity meeting aerobic physical activity guidelines only was highest among White women (24.2%) compared with Black women (18.0%) and Hispanic women (18.5%).

**TABLE 1:** Estimated prevalence of self-reported leisure-time physical activity meeting aerobic and muscle-strengthening physical activity guidelines among women aged 18–44 years, by race and ethnicity, age, and educational attainment* — National Health Interview Survey, United States, 2022 and 2024

Characteristic	Study population^†^			% (95% CI)			
	Unweighted no.	Weighted %		Meet neither	Meet both	Aerobic only	Muscle-strengthening only
**Total**	**10,891**		**100.0**	**47.2** **(46.0–48.4)**	**25.1** **(24.1–26.1)**	**21.7** **(20.7–22.6)**	**6.1** **(5.5–6.7)**
**Race and ethnicity**							
AI/AN, NH**^§^**	192		1.7	52.4 (43.7–60.9)	21.2 (14.9–29.2)	15.6 (10.1–23.1)	10.9 (6.6–17.6)
Asian, NH	869		6.8	51.5 (47.4–55.5)	20.4 (17.5–23.7)	22.2 (19.2–25.4)	6.0 (4.4–8.1)
Black or African American, NH	1,286		13.4	53.6 (50.4–56.8)	21.4 (18.8–24.2)	18.0 (15.7–20.7)	7.0 (5.5–8.7)
White, NH	5,910		53.3	42.7 (41.1–44.3)	27.6 (26.2–29.1)	24.2 (23.2–25.6)	5.5 (4.8–6.3)
Hispanic or Latino	2,369		22.3	51.9 (49.4–54.5)	23.4 (21.6–25.3)	18.5 (16.6–20.4)	6.2 (5.2–7.5)
Other single, multiple races	265		2.5	52.7 (44.9–60.4)	20.5 (15.3–26.8)	17.8 (12.7–24.5)	9.0 (5.4–14.5)
**Age group, yrs**							
18–24	1,778		25.1	44.4 (41.8–47.1)	27.8 (25.5–30.3)	22.0 (19.8–24.3)	5.7 (4.6–7.2)
25–29	1,971		18.2	46.1 (43.4–48.8)	26.2 (24.0–28.5)	22.0 (20.0–24.2)	5.7 (4.7–7.0)
30–34	2,382		19.5	49.2 (46.8–51.5)	23.2 (21.3–25.2)	20.7 (19.0–22.6)	6.9 (65.8–8.1)
35–39	2,923		22.3	48.6 (46.4–50.7)	24.2 (22.4–26.1)	21.2 (19.5–23.0)	6.1 (5.2–7.2)
40–44	1,837		14.9	48.7 (46.0–51.4)	22.7 (20.7–24.9)	22.6 (20.4–24.9)	6.0 (4.7–7.5)
**Educational attainment**							
Less than high school	729		9.0	63.2 (58.6–67.4)	13.2 (10.5–16.6)	18.6 (15.6–22.0)	5.0 (3.4–7.4)
High school diploma or GED certificate equivalent	2,288		23.7	56.3 (53.8–58.8)	17.1 (15.4–18.9)	21.3 (19.3–23.4)	5.3 (4.3–6.5)
Some college or associate degree	3,025		30.8	46.6 (44.6–48.6)	24.5 (22.8–26.3)	22.4 (20.7–24.2)	6.5 (5.5–7.7)
Bachelor's degree	3,021		23.5	38.0 (35.9–40.2)	33.4 (31.4–35.4)	22.5 (20.8–24.3)	6.1 (5.1–7.2)
Graduate degree or professional school	1,828		12.9	37.7 (35.2–40.2)	34.0 (31.7–36.4)	21.2 (19.2–23.3)	7.1 (5.9–8.6)

**Abbreviations**: AI/AN = American Indian or Alaska Native; GED = general educational development; NH = non-Hispanic.

* Physical activity guideline levels: meet neither = 0–149 minutes of moderate-to-vigorous aerobic activity and one or fewer sessions of muscle-strengthening activity per week; meet muscle-strengthening only = two or more sessions of muscle-strengthening activity and 0–149 minutes of moderate-to-vigorous aerobic activity per week; meet moderate-to-vigorous physical activity only = ≥150 minutes of moderate-to-vigorous physical activity and one or fewer muscle-strengthening activity sessions per week; and meet both aerobic and muscle-strengthening = ≥150 minutes of moderate-to-vigorous physical activity and two or more muscle-strengthening activity sessions per week.

^†^ N = 10,891 unweighted and n = 56,087,223 weighted. Data weighted to account for National Health Interview Survey complex survey design and nonresponse.

^§^ Alone or in combination with another race.

Women with higher educational attainment were more likely to report activity meeting both physical activity guidelines. One third of women with a bachelor’s (33.4%) or graduate degree (34.0%) reported activity meeting both physical activity guidelines compared with 17.1% of those with a high school diploma or equivalent and 13.2% of those who had completed less than high school.

Older women were less likely to report activity meeting both physical activity guidelines: whereas 27.8% of women aged 18–24 years reported activity meeting both physical activity guidelines, 23.2% and 22.7% of those aged 30–34, and 40–44 years, respectively, did so. The percentage of respondents who reported meeting muscle-strengthening physical activity guidelines only did not differ by demographic characteristics.

### Levels of Physical Activity Participation

An estimated 55.3% of women aged 18–44 years reported engaging in no muscle-strengthening activity (Table 2). Sufficient and insufficient muscle-strengthening activity were reported by 31.1% and 13.6% of women, respectively. High, sufficient, insufficient, and no moderate-to-vigorous aerobic physical activity were reported by 26.4%, 20.3%, 30.0%, and 23.2% of women, respectively. Women with a bachelor’s or graduate degree were more likely to report sufficient or high levels of moderate-to-vigorous aerobic physical activity than were those with a high school diploma or equivalent or less. 

**TABLE 2:** Estimated prevalence of self-reported leisure-time aerobic and muscle-strengthening physical activity among women aged 18–44 years, by level of participation, race and ethnicity, age, and educational attainment — National Health Interview Survey, United States, 2022 and 2024

Characteristic	Study population*		% (95% CI)						
	Unweighted no.	Weighted %	Moderate-to-vigorous aerobic physical activity^†^				Muscle-strengthening activity^§^		
			None	Insufficient	Sufficient	High	None	Insufficient	Sufficient
**Total**	**10,891**	**100.0**	**23.2** **(22.1–24.5)**	**30.0** **(28.9–31.2)**	**20.3** **(19.5–21.2)**	**26.4** **(25.4–27.4)**	**55.3** **(54.0–56.5)**	**13.6** **(12.8–14.4)**	**31.1** **(30.1–32.2)**
**Race and ethnicity**									
AI/AN, NH^¶^	192	1.7	25.7 (17.9–35.5)	37.5 (29.4–46.4)	14.7 (9.4–22.1)	22.1 (15.8–29.9)	55.1 (46.4–63.5)	12.8 (8.4–19.0)	32.1 (24.8–40.4)
Asian, NH	869	6.8	22.0 (18.7–25.6)	35.4 (32.0–39.0)	22.9 (19.8–26.4)	19.7 (16.8–22.9)	57.1 (53.0–61.2)	16.5 (13.7–19.7)	26.4 (23.1–30.0)
Black or African American, NH	1,286	13.4	31.1 (28.1–34.2)	29.5 (26.2–32.9)	17.2 (14.8–19.9)	22.3 (19.7–25.0)	59.9 (56.9–62.8)	11.7 (9.8–14.0)	28.4 (25.6–31.3)
White, NH	5,910	53.3	18.3 (16.9–19.7)	29.9 (28.5–31.3)	21.9 (20.7–23.2)	30.0 (28.5–31.4)	52.1 (50.4–53.8)	14.8 (13.7–15.9)	33.1 (31.6–34.7)
Hispanic or Latino	2,369	22.3	30.0 (27.6–32.5)	28.2 (26.1–30.4)	18.5 (16.6–20.5)	23.3 (21.3–25.5)	59.5 (57.1–61.9)	10.9 (9.5–12.4)	29.6 (27.6–31.7)
Other single, multiple races	265	2.5	28.8 (22.4–36.2)	32.8 (26.1–40.3)	15.8 (11.0–22.2)	22.5 (17.2–29.0)	54.6 (46.5–62.5)	16.0 (11.2–22.3)	29.4 (22.8–37.1)
**Age group, yrs**									
18–24	1,778	25.1	22.3 (20.6–25.4)	27.3 (24.8–29.9)	19.8 (17.8–21.9)	30.0 (27.7–32.5)	50.5 (47.8–53.3)	15.9 (14.0–18.0)	33.6 (31.1–36.1)
25–29	1,971	18.2	23.8 (21.6–26.1)	28.0 (25.7–30.5)	19.0 (17.2–21.0)	29.2 (26.8–31.7)	53.4 (50.8–55.9)	14.7 (12.9–16.8)	31.9 (29.5–34.4)
30–34	2,382	19.5	23.7 (21.6–26.0)	32.3 (30.2–34.6)	20.1 (18.3–21.9)	23.9 (22.0–25.9)	56.9 (54.6–59.3)	13.0 (11.6–14.5)	30.1 (27.9–32.3)
35–39	2,923	22.3	22.6 (20.7–24.6)	32.0 (30.1–34.0)	21.1 (19.3–22.9)	24.3 (22.6–26.1)	57.8 (55.6–59.9)	12.0 (10.6–13.4)	30.3 (28.3–32.3)
40–44	1,837	14.9	23.5 (21.2–26.0)	31.2 (28.7–33.8)	22.0 (19.9–24.3)	23.3 (21.2–25.5)	59.6 (56.8–62.3)	11.7 (10.1–13.5)	28.7 (26.3–31.2)
**Educational attainment **									
Less than high school	729	9.0	42.7 (38.3–47.2)	25.5 (21.9–29.4)	12.9 (10.4–16.0)	18.9 (15.8–22.5)	72.8 (68.6–76.7)	8.9 (6.8–11.7)	18.3 (15.0–22.1)
High school diploma or GED certificate equivalent	2,288	23.7	33.0 (30.6–35.6)	28.6 (26.4–30.9)	16.6 (14.9–18.5)	21.7 (19.8–23.8)	67.6 (65.3–69.8)	10.0 (8.7–11.5)	22.4 (20.6–24.3)
Some college or associate degree	3,025	30.8	22.5 (20.7–24.4)	30.6 (28.7–32.6)	19.5 (17.9–21.3)	27.4 (25.6–29.2)	55.0 (52.9–57.0)	14.0 (12.5–15.6)	31.0 (29.2–32.9)
Bachelor's degree	3,021	23.5	12.8 (11.5–14.3)	31.3 (29.3–33.2)	25.5 (23.8–27.3)	30.4 (28.5–32.3)	44.6 (42.4–46.8)	15.9 (14.4–17.6)	39.4 (37.3–41.6)
Graduate degree or professional school	1,828	12.9	12.6 (11.0–14.4)	32.2 (29.8–34.8)	24.5 (22.3–26.9)	30.6 (28.2–33.2)	40.5 (38.1–43.1)	18.3 (16.3–22.1)	41.2 (38.8–43.6)

**Abbreviations**: AI/AN = American Indian or Alaska Native; GED = general educational development; NH = non-Hispanic.

* N = 10,891 unweighted and n = 56,087,223 weighted. Data weighted to account for National Health Interview Survey complex survey design and nonresponse.

^†^ Sufficiency of aerobic moderate-to-vigorous physical activity: none = 0 minutes per week, insufficient = 1–149 minutes per week, sufficient = 150–300 minutes per week, and high = >300 minutes per week.

^§^ Sufficiency of muscle-strengthening activity: none = zero sessions per week, insufficient = one session per week, and sufficient = two or more sessions per week.

^¶^ Alone or in combination with another race.

More White women reported sufficient muscle-strengthening activity (33.1%) than did Asian (26.4%) women. Women aged 18–24 years reported the lowest prevalences of no muscle-strengthening activity (50.5%); prevalence increased with increasing age. Prevalence of sufficient muscle-strengthening activity increased with educational attainment: the highest prevalence was reported by women with more education (41.2% among those who completed graduate school), and the lowest (18.3%) was reported by women with less than a high school education.

High levels of moderate-to-vigorous aerobic physical activity were more commonly reported by White women (30.0%) than by women who were Asian (19.7%), Black (22.3%), or Hispanic (23.3%). Younger women were also more likely to report high levels of moderate-to-vigorous aerobic physical activity: 30.0% of women aged 18–24 years and 29.2% of women aged 25–29 years reported high moderate-to-vigorous aerobic physical activity, compared with 23.3% of women aged 40–44 years. Pairwise comparisons highlighted differences among groups based on race and ethnicity, age, and highest level of educational attainment (Supplementary Table).

## Discussion

Among U.S. women of reproductive age, approximately one fourth reported leisure-time activity meeting both aerobic and muscle-strengthening physical activity guidelines during 2022 and 2024. These findings align with a CDC National Center for Health Statistics data brief that found that 28.7% and 22.7% of women aged 18–34 and 35–44, respectively, met both physical activity guidelines in 2020 (*3*). Although the national prevalence of meeting physical activity guidelines has increased in recent decades (*4*), this report’s estimates suggest that significant differences in level of leisure-time physical activity exist among women of reproductive age, with differences by race and ethnicity, age, and educational attainment. White women, younger women, and those with more educational attainment reported higher prevalences of engaging in sufficient or high moderate-to-vigorous aerobic physical activity, engaging in any muscle-strengthening physical activity, and meeting both physical activity guidelines. These findings are consistent with those from previous research findings that leisure-time physical activity participation is lower among Black and Hispanic adults than among White adults and among those with less educational attainment than among those with more educational attainment (*4*). 

Some groups with lower leisure-time physical activity participation, including Hispanic and Black adults and those with less educational attainment, also experience higher rates of chronic disease–associated morbidity and mortality (*5*,*6*). Given that physical activity can help to prevent or mitigate chronic diseases (*1*), focused interventions that increase physical activity, particularly among these groups, could help reduce preventable differences in health outcomes.

Physical activity also has numerous maternal health benefits, including lowering risk of preeclampsia and gestational diabetes (*1*,*2*). Safely increasing physical activity among women of reproductive age could be an important strategy for improving these and other pregnancy-related outcomes, including reducing the length of labor and postpartum recovery (*1*,*2*). Evidence suggests most adults maintain or reduce leisure-time physical activity as they age; therefore, establishing physical activity habits during early adulthood might attenuate decreases later in life (*7*).

Approximately one half of women of reproductive age did not participate in any muscle-strengthening activities, highlighting opportunities to promote this beneficial activity. Approximately one in four women reported activity meeting aerobic physical activity guidelines only, whereas approximately one in 15 reported activity meeting muscle-strengthening physical activity guidelines only, suggesting that moderate-to-vigorous aerobic physical activity might be more accessible or prioritized over muscle-strengthening activity. This might be related to barriers specific to muscle-strengthening activity, such as accessing weights or resistance bands, knowing proper techniques, or social perceptions (*8*). Incorporating muscle-strengthening activity into existing aerobic physical activity programs might increase participation in muscle-strengthening activity.

In the United States, access to physical activity opportunities and resources varies, with marked differences in open space, safely walkable neighborhoods, group exercise opportunities, gym equipment, and resources (e.g., free time, childcare, and social support), necessitating health promotion strategies that consider social and structural factors (*9*,*10*). Active People, Healthy Nation, CDC’s national initiative to improve physical activity by 2027, promotes evidence-based strategies, including communications campaigns designed for different levels of educational attainment, to increase physical activity and address related differences in health outcomes. Tailored approaches (e.g., creating safe and convenient places for physical activity in underserved areas, including rural and low income counties, and developing walking groups or buddy systems (*1*)), might help address physical activity differences.

### Limitations

The findings in this report are subject to at least three limitations. First, this cross-sectional study uses self-reported physical activity data, which are potentially subject to recall or social desirability bias. Second, NHIS asks about leisure-time physical activity only and does not account for other types (e.g., occupational physical activity), potentially underestimating participation in physical activity. Finally, smaller sample sizes, (e.g., among American Indian and Alaska Native women) might have limited statistical power to identify differences among groups. Reliability of estimates was not assessed, and some estimates might be unstable.

### Implications for Public Health Practice

Nearly three fourths (74.9%) of U.S. women of reproductive age do not meet both physical activity guideline recommendations. Significant differences in meeting recommendations by race and ethnicity, age, and educational attainment underscore the value of addressing possible barriers to physical activity among specific groups, which could be an important strategy for improving health outcomes and reducing related differences in outcomes.

## Footnotes

* Many U.S.-based studies define reproductive age in women as 15–49 years; this study included women aged 18–44 years to be consistent with CDC reproductive health indicators (CDC’s Division of Reproductive Health works to improve mental health among women of reproductive age) and the focus on adult physical activity.

† Body composition refers to a person’s distribution of fat, bone, and muscle and is a predictor of metabolic and overall health. Physical activity can improve body composition (e.g., reduce body fat and increase muscle) and reduce risk of chronic disease independent of weight loss.

§ Data come from the Sample Adult questionnaire (National Health Interview Survey | National Health Interview Survey | CDC). Information for the final Sample Adult response rates can be found at Survey Description, National Health Interview Survey, 2022 and Survey Description, National Health Interview Survey, 2024.

¶ Respondents with less than a high school diploma or equivalent were more likely to be missing data for moderate-to-vigorous aerobic physical activity than were respondents with some college or an associate, bachelor’s, or graduate degree, and were more likely to be missing data for muscle-strengthening data than were respondents with a bachelor’s or graduate degree. Non-Hispanic Black or African American respondents were more likely to be missing data for moderate-to-vigorous aerobic physical activity and more likely to be missing data for muscle-strengthening activities than were non-Hispanic American Indian or Alaska Native, non-Hispanic Asian, Hispanic or Latino, and non-Hispanic White respondents.

** Survey prompts for aerobic activity are available at 2024 National Health Interview Survey (NHIS) Questionnaire. The 2024 prompts were the same as those in 2022. Minutes of vigorous-intensity activity were multiplied by 2, as is standard practice, to be equivalent to moderate-intensity minutes. Frequency and duration of activity were multiplied to calculate minutes per week.

†† The survey prompt for muscle-strengthening activity included the question regarding frequency of muscle-strengthening activity, “Including activities that you mentioned earlier, how often do you do leisure-time physical activities specifically designed to strengthen your muscles such as sit-ups, push-ups, or lifting weights?”

§§ Meeting physical activity guidelines: ≥150 minutes per week of moderate intensity aerobic activity, ≥75 minutes per week of vigorous intensity aerobic activity, or an equivalent combination, and two or more sessions per week of muscle-strengthening activities. Meeting aerobic physical activity guidelines only: ≥150 minutes per week of moderate intensity aerobic activity, ≥75 minutes per week of vigorous intensity aerobic activity, or an equivalent combination. Meeting muscle-strengthening physical activity guidelines only: two or more sessions per week of muscle-strengthening activities. Meeting neither: <150 minutes per week of moderate intensity aerobic activity, <75 minutes per week of vigorous intensity aerobic activity, or an equivalent combination, and fewer than two sessions per week of muscle-strengthening activities.

¶¶ Consistent with the physical activity guideline’s classification, levels of moderate-to-vigorous aerobic physical activity include: 0 minutes per week (no moderate-to-vigorous aerobic physical activity), 1–149 minutes per week (insufficient moderate-to-vigorous aerobic physical activity), 150–300 minutes per week (sufficient moderate-to-vigorous aerobic physical activity), and >300 minutes per week (high moderate-to-vigorous aerobic physical activity).

*** Levels of muscle-strengthening activities include: zero sessions per week (no muscle-strengthening activities), one session per week (insufficient muscle-strengthening activities), and two or more sessions per week (sufficient muscle-strengthening activities).

††† 45 C.F.R. part 46.102(l)(2), 21 C.F.R. part 56; 42 U.S.C. Sect. 241(d); 5 U.S.C. Sect. 552a; 44 U.S.C. Sect. 3501 et seq.
