# *Erratum:* Vol. 74, No. 41

**DOI:** 10.15585/mmwr.mm7502a5

**Published:** 2026-01-15

The report “Leisure-Time Physical Activity Among Women of Reproductive Age — United States, 2022 and 2024” contained an error. 

On page 634 in the Abstract, the fourth sentence should have read, “Overall, an estimated 25.1% of women aged 18–44 years reported leisure time activity meeting recommendations for both aerobic and muscle-strengthening physical activity, **21.7%** reported leisure time activity meeting only the aerobic activity recommendation, and 6.1% reported leisure time activity meeting only the muscle-strengthening activity recommendation.”

